# In Silico Approach in the Evaluation of Pro-Inflammatory Potential of Polycyclic Aromatic Hydrocarbons and Volatile Organic Compounds through Binding Affinity to the Human Toll-Like Receptor 4

**DOI:** 10.3390/ijerph19148360

**Published:** 2022-07-08

**Authors:** Marie Beatriz Cabral, Celine Joy Dela Cruz, Yumika Sato, Glenn Oyong, Ofelia Rempillo, Maria Cecilia Galvez, Edgar Vallar

**Affiliations:** 1 Environment and RemoTe Sensing Research (EARTH) Laboratory, Department of Physics, College of Science, De La Salle University Manila, 2401 Taft Avenue, Manila 0922, Philippines; marie_beatriz_cabral@dlsu.edu.ph (M.B.C.); celine_joy_delacruz@dlsu.edu.ph (C.J.D.C.); yumika_sato@dlsu.edu.ph (Y.S.); ofelia.rempillo@dlsu.edu.ph (O.R.); maria.cecilia.galvez@dlsu.edu.ph (M.C.G.); 2 Molecular Science Unit Laboratory, Center for Natural Sciences and Ecological Research, De La Salle University, 2401 Taft Avenue, Manila 0922, Philippines; glenn.oyong@dlsu.edu.ph

**Keywords:** in silico, molecular dynamics, TLR4, PAHs, VOCs, binding affinity

## Abstract

Polycyclic aromatic hydrocarbons (PAHs) and volatile organic compounds (VOCs) are widespread across the globe, existing in the environment in complex mixtures potentially capable of initiating respiratory illnesses. Here, we use an in silico approach to evaluate the potential pro-inflammatory effects of various carcinogenic PAHs and VOCs through their binding affinity towards the human toll-like receptor 4 (TLR4). For receptors and ligands, RCSB Protein Data Bank and PubChem were used in obtaining their 3D structures, respectively. Autodock Vina was utilized to obtain the best docking poses and binding affinities of each PAH and VOC. Out of the 14 PAHs included in this study, indeno(1,2,3-cd)pyrene, benzo(ghi)perylene, and benzo[a]pyrene had the highest binding affinity values of −10, −9, and −8.9 kcal/mol, respectively. For the VOCs, out of the 10 compounds studied, benzene, 1,4-dichlorobenzene, and styrene had the highest binding affinity values of −3.6, −3.9, and −4.6 kcal/mol, respectively. Compounds with higher affinity than LPS (−4.1 kcal/com) could potentially induce inflammation, while compounds with lower affinity would be less likely to induce an inflammatory response. Meanwhile, molecular dynamics simulation and RMSF statistical analysis proved that the protein, TLR4, stably preserve its conformation despite ligand interactions. Overall, the structure of the TLR4 was considered inflexible.

## 1. Introduction

TLR2 and TLR4 are toll-like receptors that are involved in the innate immune system. Both identify main bacterial components, namely lipoproteins in TLR2 and lipopolysaccharides (LPS) in TLR4. TLR2, TLR4, and TLR7 are the best characterized pattern recognition receptors (PRRs), which identify invading pathogens outside the cell as well as intracellular pathogens captured in endosomes [1,2,3]. TLR4 activation occurs through dimerization of the receptor where it forms a homodimer. When LPS binds to the lipopolysaccharide-binding protein (LBP), TLR4 is activated [4]. LPB is a soluble shuttle protein that binds to LPS and directly facilitates the association of LPS and CD14 [5]. Several transmembrane proteins are essential for the TLR signaling pathway. CD14, a glycophosphatidylinositol-anchored protein, serves as the co-receptor with TLR4. While myeloid differentiation factor-2 (MD-2) serves as the LPS recognition site [6]. MD-2 is a soluble protein that non-covalently binds with TLR4. However, in the absence of TLR4, MD-2 can directly form a complex with LPS. After the LPS is recognized, TLR4 undergoes oligomerization and induces downstream adaptors through interactions with the TIR (Toll-interleukin-1 receptor) domains. The TIR domains include five main adapter proteins: MyD88 (myeloid differentiation primary response gene 88), TIRAP (TIR domain-containing adaptor protein), TRIF (TIR domain-containing adaptor inducing IFN-β), TRAM (TRIF-related adaptor molecule), and SARM (sterile α and HEAT-Armadillo motifs-containing protein) [5]. TLR4 signaling takes place through one of two distinct intracellular routes. TIRAP promotes the transcription factor nuclear factor-κB (NF-κB), which results in the production of pro-inflammatory cytokines such as interleukin-6 (IL-6) and tumor necrosis factor-α (TNF-α). Alternatively, through TRAM and TRAF, the MyD88-independent route results in the release of type 1 interferons. Research has shown that actual cellular localization of TLRs is critical in signaling control, and that cell-type-specific signaling downstream of TLRs affects certain innate immune responses [6].

Polycyclic Aromatic Hydrocarbons (PAHs) are groups of dietary and environmental contaminants that are highly diverse. PAHs are pervasive environmental contaminants formed mostly by the incomplete combustion of coal, oil, petrol, and wood. They arise through insufficient kindling of organic matter and exist in the environment in the form of complex mixtures, in gaseous form, and in particulate matter (PM), as well as in soil particles and sediments. They also contribute to certain health disorders correlating to immunotoxicity, endocrine disruption, or tumor growth [7]. PAHs are aromatic hydrocarbons that contain more than one benzene ring and do not contain a heteroatom. PAHs with less than five rings are referred to as light PAHs, while those with five or more rings are known as heavy PAHs. These compounds are mainly composed of oxygen and hydrogen that are bound in simple to complex ring systems, in which the arrangement of the rings varies its physical, chemical, and toxicological properties [8]. Furthermore, in assessments of the cancer risk posed by PAHs in the atmosphere, scientists and regulators rarely take into account the complex mixture of emitted compounds and degradation products, and they frequently represent the entire mixture using a single emitted compound, benzo[a]pyrene. Nearly 90 percent of cancer risk globally arises from other PAHs, including uncontrolled degradation products of emitted PAHs. Benzo[a]pyrene alone is an inadequate indication of PAH risk distribution and management. Using an animal-based technique, the relative contribution of various PAHs to incremental life cancer risk was compared. The other emitted PAHs account for 72 percent of the estimated worldwide human cancer risk, whereas the 12 N-PAHs (6 nitro-PAHs and 6 dinitro-PAHs) are known to account for the remaining 17 percent of the computed global additional life cancer risk [9]. Moreover, according to Richard [10], each year in the UK, an estimated 28,000–36,000 individuals lose their lives as a consequence of air pollution. This is a considerable rise from 2015, when the number was around 29,000. When gasoline and other fuels are burned, nitrogen dioxide and fine particle pollution are created. In addition, the effects of other pollutants such as fine particulate matter, PAHs, and VOCs also contribute invariably to the number of fatalities.

Volatile organic compounds (VOCs) are present in a wide variety of consumer items, including furniture, sealants, and paints. VOCs have been connected to lung disorders due to their high degree of reactivity with the airway epithelium and mucosa. VOCs may cause a variety of health problems when inhaled. Propylene glycol and glycol ethers (PG), benzene, and formaldehyde are just a few of the VOCs that are known to react strongly with the respiratory tract’s epithelial lining and mucous membrane. Additionally, exposure to VOC is related to an increased risk of developing asthma and other respiratory illnesses. Although particulate matter exposure results in oxidative stress, VOC exposure results in a substantial degree of chemical reactivity with the amine-rich epithelial lining and mucosal membrane through a C–O polar bonding mechanism, resulting in inflammation. Thus, VOC mitigation solutions must take the chemical makeup of VOCs into account [11]. In urban environments, hazardous VOCs and solvents readily combine with nitrogen oxides (NOx) to generate ozone (O3) through a series of photochemical reactions, resulting in a photochemical haze in the troposphere. Significantly, several VOCs are listed as hazardous air pollutants due to their potential to produce harmful human health impacts such as cancer and respiratory disease [12].

**Figure 1:**
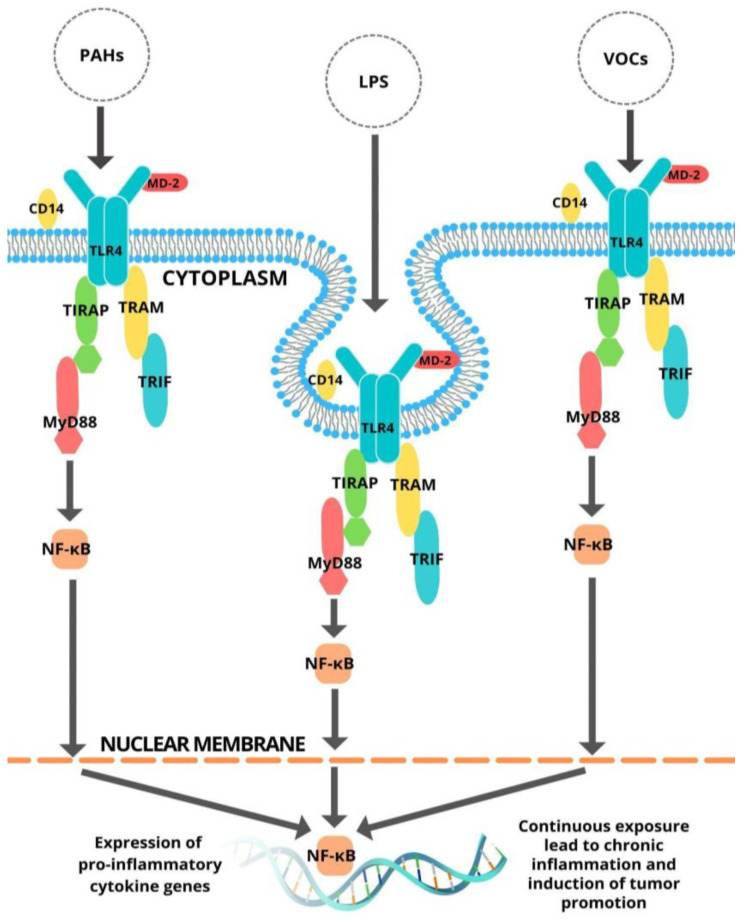
Plausible mechanism for TLR4 signaling pathway activation by LPS, PAHs, and VOCs.

Lipopolysaccharide (LPS), a component of the cell wall of gram-negative bacteria, is widely regarded as a significant pathogenic component in bacterial infection and can eventually induce systemic inflammatory response syndrome. In response to infection, the immune system produces proinflammatory mediators such as IL-6 and TNF-α, but excessive production will lead to systemic inflammatory response syndrome. LPS triggers macrophages or monocytes through TLR4. LPS is recognized by TRL4 along with additional molecules like CD14 and MD-2 [13]. LPS is made up of a variable polysaccharide domain that is covalently bonded to a diglucosamine-based acylated phospholipid called lipid A. Despite the fact that the polysaccharide content of LPS varies greatly, the lipid A domain is substantially conserved. Purified and synthesized lipid A preparations display significant LPS agonist action; consequently, it appears that lipid A domain is responsible for the majority of LPS’ pharmacological activities [14]. The mechanism of the TLR4 signaling pathway by LPS and various PAHs and VOCs is shown in Figure 1. 

The contact of a chemical molecule with a protein may trigger a biological activity. This encompasses the stimulation or inhibition of an enzyme’s activity in addition to the impact of a pharmaceutical substance on its target protein. Binding affinity measures the force with which a binding molecule, sometimes called a ligand, attaches to its matching protein [15]. The binding energies of optimally docked molecules varied from –8.0 to –11.71 kcal/mol [16]. Moreover, affinities closer to –10 kcal/mol are an indication of efficient binding, whereas affinities of less than or equal to –5 kcal/mol depict negligible binding [17].

Carcinogens are chemicals that contribute to the formation of cancer, which may be described as the uncontrollable growth of neoplastic cells. Cancer can be caused by a variety of factors. Any one of the roughly 100 trillion cells that make up a human body has the potential to be changed into a cancerous or malignant cell by a number of different agents. These agents could have a chemical make-up, a biological make-up, or a physical make-up [18]. The identification of tumorigenic potential in animals and the evaluation of the related risk in human subjects are the two primary goals of carcinogenicity investigations [19]. When PAHs form a strong bond with the TLR4 protein, this interaction may lead to a bigger impairment of immunity and pathogenesis, both of which may contribute to the development of cancer most especially by persistent exposure. The TLR-dependent signaling pathway is altered as a result of PAH exposure. As a consequence of alterations in the signaling pathway, altered cytokine profiles are produced, which in turn leads to an increased proinflammatory response in the respiratory (inhaled) or gastrointestinal (ingested) tracts. PAHs have the potential to be biotransformed into chemically reactive intermediates, which then have the potential to establish covalent bonds with biological macromolecules. This may be the first step in the development of mutations and cancers [20]. In recent studies, the binding affinity of dibenzo[a,l]pyrene-11,12-diol with CYP1A1 and CYP1B1 was computed with the use of AutoDock Tools. With a relatively higher affinity, it can be an indication of their involvement in carcinogenic activity [21].

In light of the fact that in silico methods, such as molecular docking, are acknowledged to possess faster procedural turnover with a wide range of accessibility to other bioinformatics tools, applying such methods in medicine and healthcare studies has gained popularity. In comparison to conventional approaches to cancer research, the cost and amount of time spent on the investigation of the carcinogenic potential of PAHs and VOCs are practically more competitive. Because it makes use of high-throughput molecular docking, the in silico approach made it possible to choose the best-docked ligands from among a vast array of compounds. In aid of deeper understanding, the findings of this study have assisted in the assessment of the pro-inflammatory potential of PAHs and VOCs via the TLR4 pathway, the information of which can be used as baseline to raise awareness among health enthusiasts, organizations, and regular citizens. Furthermore, this may serve as a reference for researchers exploring similar topics. With sufficient knowledge regarding the pro-inflammatory effects of PAHs and VOCs, the results of the research have provided a better grasp on how to handle and reduce the risks of inevitable exposure to the aforementioned substances and to facilitate confirmatory wet laboratory or in vivo experiments in the future.

The focus of this study is the potential pro-inflammatory effects of 14 PAHs and 10 VOCs via TLR4 using an in silico approach. The selected compounds are considered carcinogens or possibly carcinogenic to human health. Autodock Vina v.1.2.0 (Scripps Research, San Diego, CA, USA) was used to compute the binding affinity of the carcinogens with the TLR4 receptor.

## 2. Materials and Methods

The plausible mechanism of the activation of the TLR4 signaling pathway by various PAHs and VOCs, and LPS is shown in Figure 1. Fourteen (14) PAHs were identified by the United States Environmental Protection Agency (US EPA) as priority pollutants on the basis of being reported with the highest concentrations, having the greatest exposure to human health, recalcitrant nature, and toxicity [22]. Additionally, 10 VOCs classified by the World Health Organization (WHO) as carcinogenic or possibly carcinogenic to humans were also included in the study [23].

### 2.1. Preparation of ReceptorProtein

The human TLR4 served as the receptor. The 3D structure was downloaded as sdf file (PDB ID: 4G8A) and converted to pdb via Open Babel v.2.4.0 (GPL v2, SourceForge, San Diego, CA, USA, https://sourceforge.net/projects/openbabel/, accessed on 2 June 2022). Using the BIOVIA Discovery Studio Visualizer v.21.1 (Dassault Systèmes, Waltham, CA, USA), water and other heteroatoms were removed from the molecule and the binding site was defined from a single subunit with xyz center (−22.061059, −17.924471, and −17.924471) and size coordinates (10, 10, 10) respectively. The protein was finally prepared for docking through the addition of polar hydrogen and Koolman charges using AutoDockTools (ADT) v.1.5.6 (Scripps Research, CA, USA) and saved as pdbqt. Additionally, the stability and reliability of the prepared protein were examined using the following; Zlab (UMass Chan Medical School, Worcester, MA, USA, https://zlab.umassmed.edu/bu/rama/, accessed on 2 June 2022), and Verify 3D and ERRAT (UCLA-DOE, Los Angeles, CA, USA, https://saves.mbi.ucla.edu/, accessed on 2 June 2022) servers.

### 2.2. Preparation of Ligands

The sdf files for the 3D structures of the ligands were obtained from PubChem. Using OpenBabel, the sdf files of the 14 PAHs and 10 VOCs were converted to pdbqt. The 14 PAHs are as follows: naphthalene (CID:6195), acenaphthene (CID:139069599), acenaphthylene (CID:9161), fluorene (CID:6853), phenanthrene (CID:995), anthracene (CID:8418), fluoranthene (CID:9154), pyrene (CID:31423), benzo[a]anthracene (CID:5954), chrysene (CID:9171), benzo[k]fluoranthene (CID:9158), benzo[a]pyrene (CID:2336), benzo(ghi)perylene (CID:9117), and indeno(1,2,3-cd)pyrene (CID: 9131), while the 10 VOCs are methylene chloride (CID:6344), vinyl chloride (CID:6338), 1,2-dichloroethane (CID:11), chloroform (CID:6212), trichloroethylene (CID:6575), tetrachloroethylene (CID:31373), 1,3-butadiene (CID:7845), benzene (CID:241), 1,4- dichlorobenzene (CID:4685), and styrene (CID:7501). Additionally, LPS (CID:11970143) was also obtained from PubChem and served as the basis for comparison of the binding affinity. All structures of PAHs, VOCs, and LPS in pdbqt format were compiled in a folder to be used for molecular docking later on.

### 2.3. Preparation of Docking Configuration File

In a new and separate folder, the pdbqt files for the receptor and the ligands were copied along with the AutoDock Vina programs following the recommended procedure. Before actual docking, preparation of the docking configuration file (conf.txt) was created using Windows notepad. The input file in text format contained the receptor (TLR4), the ligand, and the xyz size and center coordinates. The configuration file for each ligand was saved in the same folder.

### 2.4. Molecular Docking

Intermediary tasks, such as the production of pdbqt files for the TLR4 protein and ligands and the building of grid boxes with the use of the graphical user interface ADT were double checked for the TLR4 protein (polar hydrogens, Kollman charges on joined atoms, solvation parameters, and fragmental volumes). AutoDock Vina v.1.2.0. (Scripps Research, CA, USA) was used to perform docking, utilizing the protein and ligand information from the configuration file, as well as grid box parameters. Exhaustiveness was set at 8. TLR4 was treated as stiff throughout the docking phase. The findings with a positional root-mean-square deviation (RMSD) less than 1.0 were clustered together and represented by the result with the lowest binding free energy. For additional study, the docking pose with the lowest binding energy was extracted and matched with the TLR4 receptor structure [24] and visualized using BIOVIA Discovery Studio.

### 2.5. Molecular Dynamics Simulation

All molecular dynamics simulations (MDS) were performed using the CABS-flex 2.0 server (http://biocomp.chem.uw.edu.pl/CABSflex2, accessed on 2 June 2022) to validate the stability of the complex and evaluate the conformation changes in the protein. The initial structure files for the apo (unbound) structure and the bound structures of TLR4 were uploaded in pdb formats. The raw data for the fluctuation plot of the apo structure as well as the structure for each TLR4 bound to PAH and VOC were transferred into a MATLAB sheet. The 2D data were plotted using MATLAB R2021a (MathWorks, Natick, MA, USA), wherein the RMSF of each amino acid is plotted against the residue number. Meanwhile, for the statistical analysis, paired *t*-test (Microsoft Excel 2010) was applied wherein the significance value was set at 0.05. With this, the *p*-values of each chain within the TLR4 are further interpreted; *p*-values that are lower than 0.05 are an indication of significantly different fluctuations in the apo and the bound structures of TLR4.

All the MDS-generated structural models of TLR4 from 0 to 10 nanoseconds were superimposed using BIOVIA Discovery Studio Visualizer. The same program was used for the superimposed image of the bound structure to further visualize the effects of the difference after the ligand binding. From 0 to 10 nanoseconds, the models of the apo structure were overlapped with the pdbqt file of the ligand with the most stable complex interaction with the receptor.

## 3. Results

### 3.1. Preparation of the TLR4 Receptor

The quality of the prepared human TLR4 was assessed to verify its reliability and suitability in carrying out further molecular docking simulations [25]. The structural validation of the target model for the receptor was performed using online quality structural assessment tools, Zlab, Verify 3D, and ERRAT.

**Figure 2:**
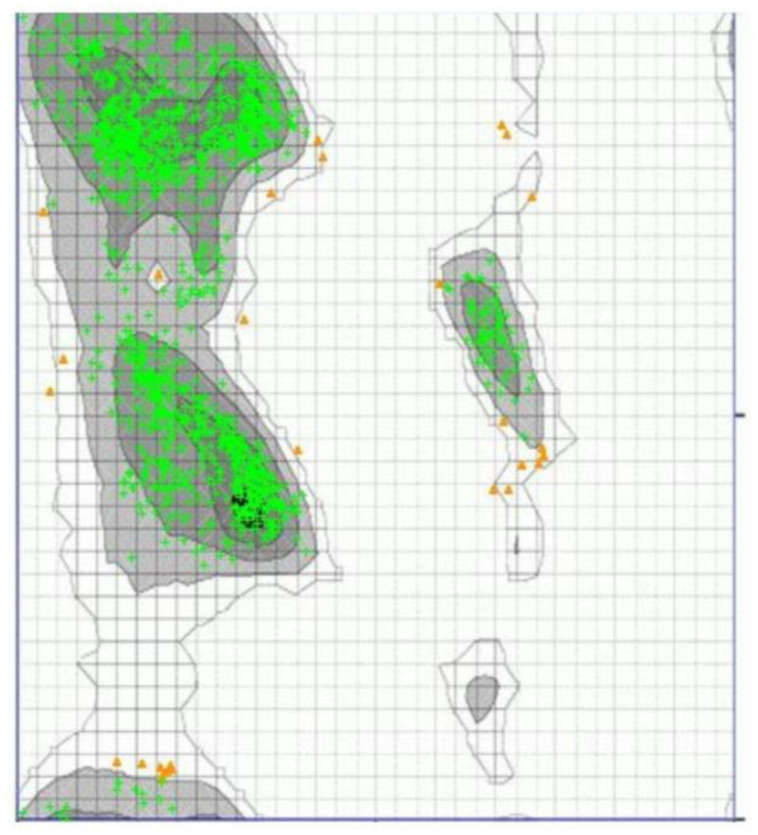
Ramachandran plot by Zlab. The gray regions are indicative of favorable regions while the areas enclosed by the line connote allowable regions. Green crosses are within the highly preferred regions while the orange triangles are within the allowed observations.

The stereochemical properties of the TLR4 molecule were evaluated using Zlab. The software generated a Ramachandran plot which depicts the distribution of the phi (φ) and psi (ψ) angles. Based on Figure 2, 98.018% of the amino acid residues (green crosses) are within the highly preferred region (dark gray areas), while only 1.982% (orange triangles) were outside the preferred regions. The phi and psi backbone dihedral angles in the protein model are reasonably accurate considering that some of the residues are within the allowed region. Additionally, no outliers (0.0%) were observed from the same plot.

**Figure 3:**
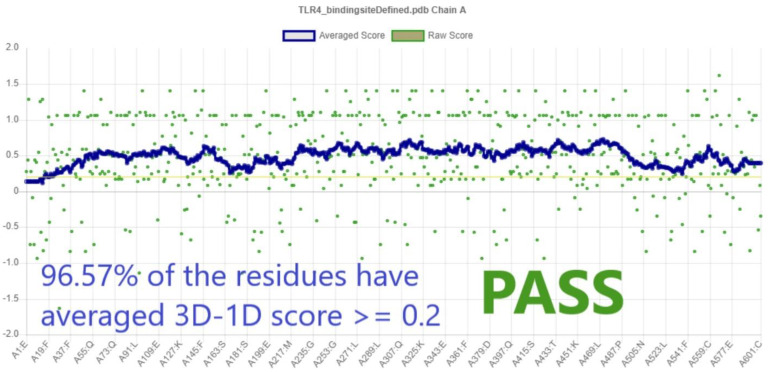
VERIFY 3D plot showing a PASS score of 96.57% with averaged 3D−1D score ≥ 0.2.

The compatibility of the TLR4 3D model with its sequence was measured using VERIFY 3D [26]. The VERIFY 3D (Figure 3) assigned a 3D–1D score of ≥0.2 for the modeled protein, and a passing score of 96.57%. This implies that the 3D model is accurate based on the folding prediction imposed by its sequence [27].

**Figure 4:**
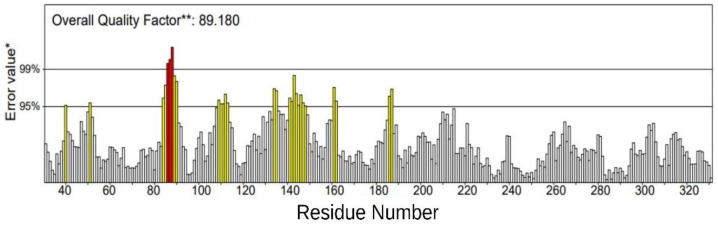
ERRAT residues with a factor of 89.180%. Yellow bars indicate residues that exceed the 95% rejection limit. Red bars indicate residues that exceed the 99% rejection limit. Grey bars indicate residues within the accepted region. (*) On the error axis, the lines indicate the 95% and 99% rejection regions. (**) Expressed as the percentage of the protein in which the calculated error value falls below the 95% rejection limit.

The overall quality factor for non-bonded atomic interactions between various atoms within the modeled structure was evaluated using ERRAT. The ERRAT score should at least be 50% to be considered a model of good quality [25]. In Figure 4, the overall quality of the model was verified at 89.180%. This represents the percentage of the protein for which the calculated error value is within the acceptable region of the 95% rejection limit.

Overall, the structure quality assessment made using the online tools. Zlab, VERIFY 3D, and ERRAT, suggested that the prepared human TLR4 is a suitable model for molecular docking simulations. From the Ramachandran plot (Figure 2), 98.018% of residues were inside the allowed regions. Moreover, the VERIFY 3D score (96.57%) (Figure 3) and ERRAT score (89.180%) (Figure 4) strongly supported the overall quality and compatibility of the 3D structure of the protein model.

### 3.2. Binding Affinity of Polycyclic Aromatic Hydrocarbons

The binding affinity, or docking score in units of kcal/mol was automatically calculated by Autodock Vina based on the total number of pairs of atoms that may move relative to one another or which can be identified as effects of intermolecular and intramolecular forces of attraction. The more negative the binding affinity, the greater the binding stability [28].

PAHs’ carcinogenicity is linked to the number of benzenoid rings in the molecule. As the number of rings increases, the carcinogenic effect also increases. As listed by the United States Environmental Protection Agency (USEPA), 11 of which are carcinogenic or mutagenic PAHs and are further classified based on carcinogenicity. For its well-known carcinogenic properties, benzo(a)pyrene has frequently been used as a guide for the carcinogenicity of other PAHs, and the majority of researchers have used benzo(a)pyrene. It should be noted that in addition to benzo(a)pyrene, research on other PAHs is also necessary [29].

The human TLR4 is considered as one of the most efficient innate immune receptors in the body which induces a proinflammatory response after it binds to a ligands with known pathogen associated molecular patterns [30]. Table 1 shows the tabulated affinities obtained through the in silico analysis of PAHs. Results have shown that the top three highest binding affinities were afforded by indeno(1,2,3-cd)pyrene, benzo(ghi)perylene, and benzo[a]pyrene. The highest of which comes from indeno(1,2,3-cd)pyrene with −10.0 kcal/mol and is classified to be highly carcinogenic. While the second and third highest have the values of −9.0 kcal/mol and −8.9 kcal/mol which are classified as moderately and highly carcinogenic, respectively. Hence, with the computed binding affinities of the three PAHs using AutoDock Vina and its presence in different environmental sources, these PAHs have the potential to induce proinflammatory effects.

### 3.3. Binding Affinity of Volatile Organic Compounds

Interactions between proteins are orchestrated in a precise and time-dependent manner, underlying cellular function. The binding of two proteins can be viewed as a reversible and rapid process in an equilibrium that is governed by the law of mass action. The binding affinity is the strength of the interaction between two (or more than two) molecules that bind reversibly [31].

Each docking experiment often results in the production of a library of ligands, which includes known inhibitors and known decoys. After a docking run is complete, the ligands are ranked according to their binding affinity to the target site, allowing for comparisons between the ligands. AutoDock Vina displays binding affinity in kcal/mol; the more negative the binding affinity, the better the fit within the binding pocket [32].

Styrene, 1,4-dichlorobenzene, and benzene have the highest binding affinity among the 10 VOCs reported in Table 2. Styrene has the highest value of −4.6 kcal/mol and is categorized as probably carcinogenic to people, while 1,4-dichlorobenzene and benzene have values of −3.9 kcal/mol and −3.6 kcal/mol, respectively, and are classified as possibly carcinogenic to humans.

### 3.4. Molecular Docking of TLR4 and Lipopolysaccharide

TLR4/MD-2 complex found on the cell surface of target cells is responsible for the attachment of LPS and activation of NF-κB downstream signaling pathway. The best-docked scoring pose as determined by AutoDock Vina was selected and visualized by using BIOVIA Discovery Studio Visualizer [33].

**Figure 5:**
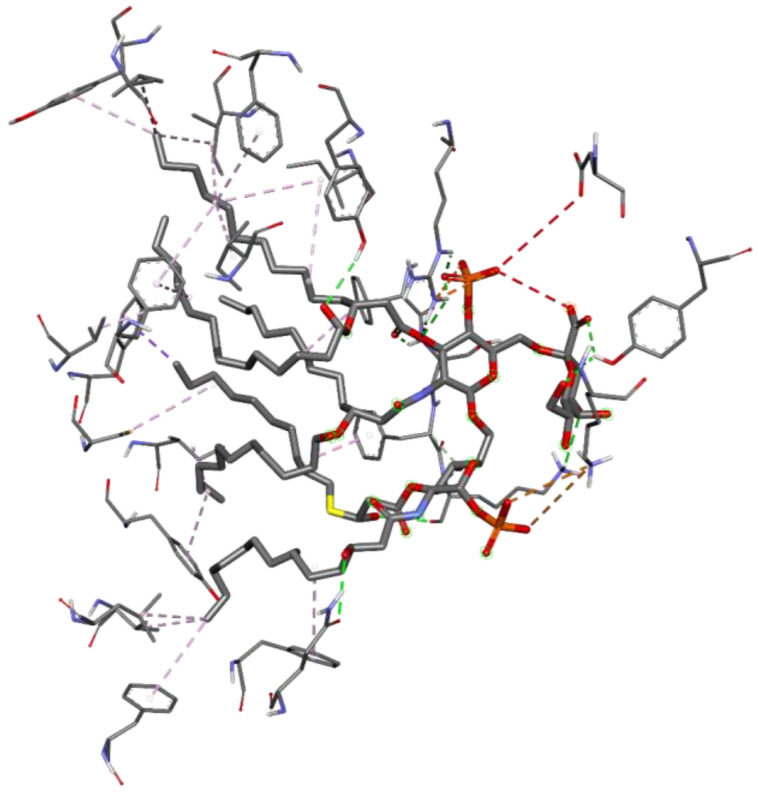
3D interaction between lipopolysaccharide and human TLR4.

The stimulation of TLR4 by LPS modulated pathways involves inflammation, permeability, and coagulation. Besides its inflammatory reactions, it can also mediate changes in VOC which have the potential to be used as biological markers [34]. Figure 5 shows the interaction of LPS with human TLR-4 which is held together by 40 molecular interactions. Furthermore, the specific interactions that between the ligand and the receptor are hydrophobic, hydrogen bond, and electrostatic interactions, while the common amino acid residues involved are Arg-264, Lys-341, Tyr-296, Tyr-102, Ser-120, Lys-122, Gln-436, Phe-121, Ile-63, Ile-94, Ile-117, Cys-133, Val-135, Ile-153, Val-82, Leu-71, Phe-440, Phe-463, Tyr-65, Phe-76, Phe-104, Phe-119, and Tyr-131. Most of the amino acid side chains such as Val, Iso, Leu, Phe, and Tyr are hydrophobic, thus interacting through hydrophobic interactions. On the other hand, amino acid residues such as Ser and Gln have polar and uncharged side chains, hence interacting via multiple hydrogen bonds. Residues with electrically charged side chains such as Arg and Lys prefer to interact through electrostatic interactions. Moreover, based on the result from the in silico experiment using Autodock Vina, the binding affinity of LPS with TLR4 is −4.1 kcal/mol.

### 3.5. Molecular Docking Interaction of Human TLR4 with PAH

Toll-Like Receptors (TLRs) may play a crucial role in pathogen recognition, innate immune activation, and malignancy in human esophageal epithelial cells. The binding affinity of PAHs to TLR4 was evaluated using AutoDock Vina [20]. Furthermore, this section is divided into four subsections that discuss different interactions between the human TLR4 and PAHs or LPS.

#### 3.5.1. Interaction of Human TLR4 Residues and PAH

Life forms rely heavily on molecular recognition, which pertains to the ability of biomolecules to recognize one another or other tiny molecules with great specificity and affinity. Proteins, a significant class of biological molecules, carry out their activities via binding to other components or to themselves [35]. Most interactions of the human TLR4 residues to different PAHs are hydrophobic in nature and vary in different types of interactions. This is entirely due to the hydrophobic nature of PAHs.

**Figure 6:**
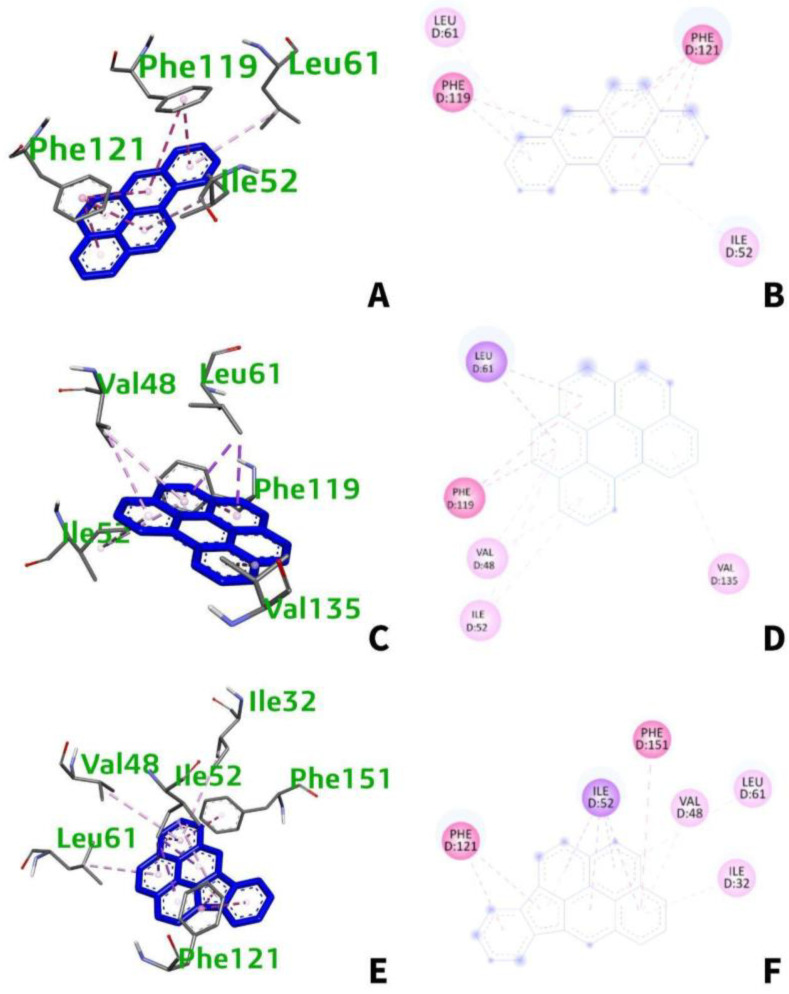
(**A**) 3D and (**B**) 2D interaction of benzo[a]pyrene with TLR-4; (**C**) 3D and (**D**) 2D interaction of benzo[ghi]perylene with TLR4; (**E**) 3D and (**F**) 2D interaction of indeno(1,2,3-cd)pyrene with TLR4.

From Table 3, benzo[a]pyrene interacts with four amino acids namely: Phe-119, Phe-121, Ile-52, and Leu-61 (Figure 6A,B). Meanwhile, benzo(ghi)perylene and indeno(1,2,3-cd)pyrene interacts with five (Leu-61, Phe-119, Val-48, Ile-52, Val-135) and six (Ile-52, Phe-121, Phe-151, Leu-61, Ile-32, Val-48) amino acids, respectively (Figure 6C,E). All interactions are hydrophobic and happen at chain D at certain distances ranging from 3.390 to 5.866 Å. The types of interactions vary from pi–pi stacking, pi–pi T-shaped, pi–pi sigma, and pi–alkyl interactions.

#### 3.5.2. Intermolecular Forces of Attraction

**Figure 7:**
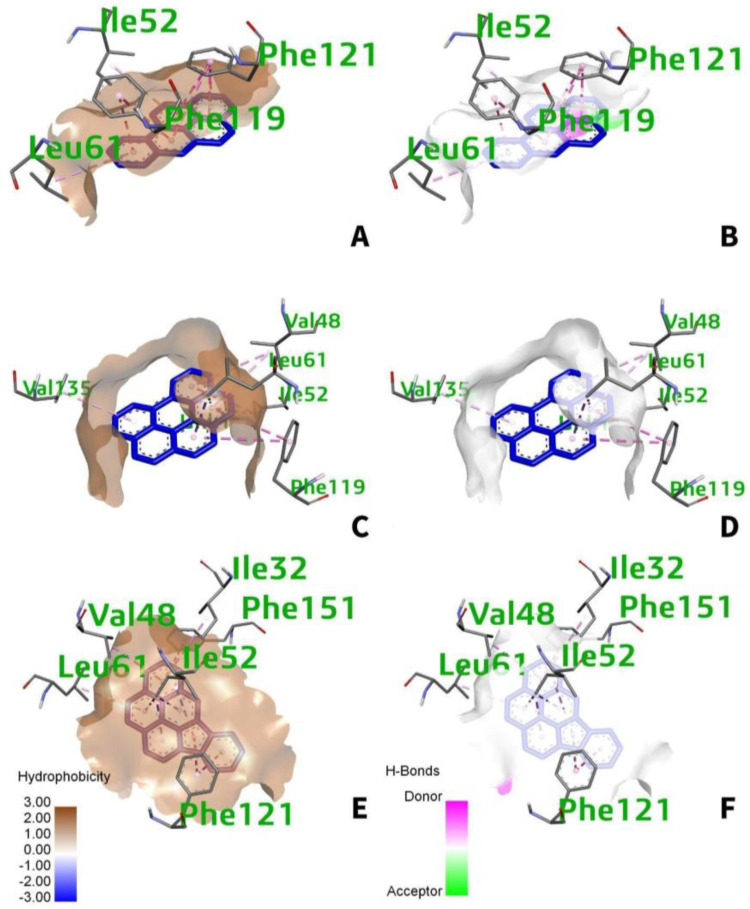
(**A**) 3D hydrophobic and (**B**) 3D H−bonding surfaces for benzo[a]pyrene; (**C**) 3D hydrophobic and (**D**) 3D H−bonding surfaces for benzo[ghi]perylene; (**E**) 3D hydrophobic and (**F**) 3D H−bonding surfaces for indeno(1,2,3-cd)pyrene. The hydrophobicity cloud is represented by the brownish color, while the hydrogen donor and acceptor regions are represented by the pink and green colors, respectively.

Almost all of the static and dynamic features of matter, such as their presence in solid, liquid, and gaseous forms, relative stability, and chemical reactivity, are regulated by intermolecular forces, which are defined by the fundamental balance of interacting components of physical and chemical nature [36]. The intermolecular forces of attraction between the human TLR4 and the individual PAH are mainly van der Waals (hydrophobic). For benzo[a]pyrene (Figure 7A), a greater predominance of the van der Waals interaction occurs within the amino acid residues, Phe-119, Phe-121, Ile-52, and Ile-61, with hydrophobic side chains. Moreover, the interactions for benzo[ghi]perylene occur at five different amino acid residues with hydrophobic side chains namely, Leu-61, Val-48, Phe-119, Ile-52, and Val-135 (Figure 7C). While for indeno(1,2,3-cd)pyrene (Figure 7E), it occurs at Phe-121, Phe-151, Ile-52, Leu-61, Ile-32, and Val-48. Most interactions between the active site of the receptor are of the van der Waals, through the visualization of the hydrophobicity cloud. However, white color surfaces indicated no presence of hydrogen bonding occurs in the region occupied or at near proximity (Figure 7B,D,F).

#### 3.5.3. Ramachandran Plot 

The Ramachandran plot, which includes the phi and psi angles, is essential for predicting the conformational space of an amino acid in a polypeptide using the hard-sphere model that represents steric coupling effects. Without the participation of the phi and psi angles, steric hindrances significantly constrain the protein’s structure. Observing the conformation of a structure was discovered empirically by plotting it in the phi–psi space. The integrity and validity of a protein’s 3D structure are determined by the conformational phi- and psi-derived spaces using the Ramachandran plot [37].

**Figure 8:**
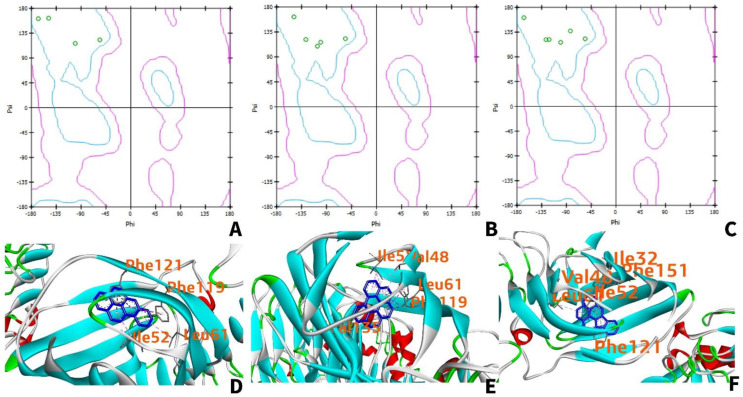
Ramachandran plots of TLR4 amino acids interacting with benzo[a]pyrene (**A**), benzo[ghi]perylene (**B**), and indeno(1,2,3-cd) pyrene (**C**). The green circles indicate the amino acid residues interacting with the ligand. The teal regions are indicative of favorable regions while the areas enclosed by the teal and pink regions are allowed but rare due to the torsion angles. Beyond the pink region, residues are not allowed due to the possibility of collision; 3D interaction of TLR4 with benzo[a]pyrene (**D**), benzo[ghi]perylene (**E**), and indeno(1,2,3-cd)pyrene (**F**).

For benzo[a]pyrene, benzo[ghi]perylene, and indeno(1,2,3-cd)pyrene, all the amino acid residues are located within the favored region (Figure 8A–C). Furthermore, residues located in the upper left corner of the Ramachandran plot are considered to be in beta-pleated sheets, while residues at the lower-left corner are right-handed α-helix, and residues located in the upper right corner are left-handed α-helix. Thus, all the amino acid residues that interact with each PAH are in beta-pleated sheet conformation. As seen in Figure 8D–F, the teal-colored beta-pleated sheet structure of the human TLR4 is mainly the region where interaction with the ligand occurs.

#### 3.5.4. TLR4 Amino Acids Interacting with LPS and PAHs

**Figure 9:**
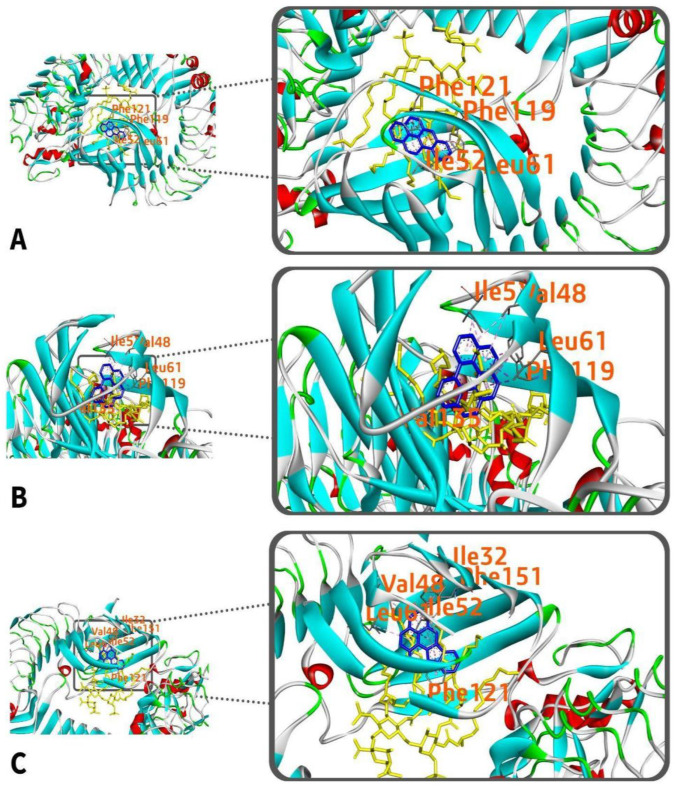
Superimposed interactions of benzo[a]pyrene (**A**), benzo[ghi]perylene (**B**), and indeno(1,2,3-cd)pyrene (**C**) with human TLR4-LPS complex. The PAH is represented by the blue molecule, while the LPS is represented by the yellow molecule.

Human TLR4 interacts hydrophobically with LPS at 40 amino acid residues (Figure 5). Table 4 shows the interaction of the common amino acid residues of the receptor to LPS and individual PAH. In contrast to the amino acids with which LPS interacts through TLR4, two of the four amino acids in benzo[a]pyrene (Figure 9A), namely Phe-119 and Phe-121, are likewise hydrophobically associated with chain D at distances of 5.369 Å and 3.974 Å, respectively. It does, however, interact through pi–alkyl interactions. While benzo[ghi]perylene and LPS share two amino acid residues (Phe-119 and Val-135) (Figure 9B). Additionally, Phe-119 interacts hydrophobically with alkyl at a distance of 5.369 Å, while Val-135 interacts hydrophobically with alkyl at a distance of 4.850 Å. Similarly, indeno(1,2,3-cd)pyrene and LPS share two amino acid residues (Phe-121 and Phe-151) (Figure 9C). Furthermore, Phe-121 interacts hydrophobically at a distance of 3.092 Å, while Phe-151 engages through a pi–sigma interaction at a distance of 3.840 Å.

Of the three PAHs investigated, two amino acid residues were shared between each ligand and LPS. The shared amino acid residues vary from Phe-121, Phe-119, Phe-151, and Val-135. Hydrophobic interactions were found to occur at chain D.

### 3.6. Molecular Dynamics Simulation of TLR4-PAH Complexes

MDS are performed in order to confirm the flexibility and dynamics of the modeled protein. The amino acid residues with the highest degree of flexibility were predicted with the help of the CABS-Flex server [38]. This section is subdivided into two subsections where discussion of the RMSF graphs and MDS are further analyzed.

#### 3.6.1. RMSF of Apo-TLR4 and TLR4-PAH Complexes

The value of the root mean square fluctuations (RMSF) obtained from CABS-Flex are indicative of high residue flexibility [38]. RMSF is calculated from trajectories, which is a characteristic of the flexibility of each amino acid residue in the protein [39]. Here, the RMSF was plotted using MATLAB R2021a.

**Figure 10:**
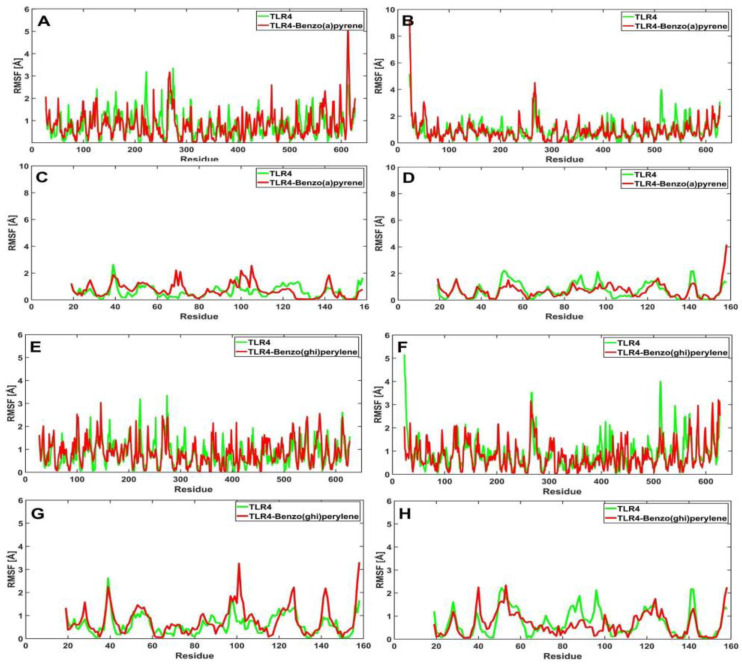

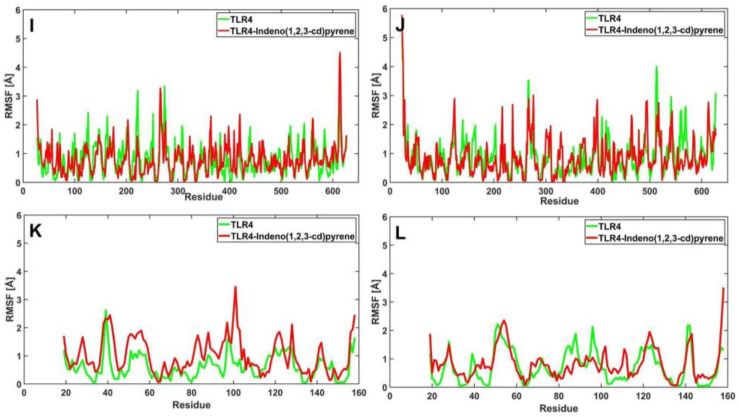
RMSF profiles for the amino acid residues of the apo (green) and bound (red) structures of TLR4: TLR4 vs. TLR4-benzo[a]pyrene chains A–D (**A**–**D**); TLR4 vs. TLR4-benzo(ghi)perylene chains A–D (**E**–**H**); and TLR4 vs. TLR4-indeno(1,2,3-cd)pyrene chains A–D (**I**–**L**).

TLR4 bound to benzo[a]pyrene (Figure 10A–D) shows minimal fluctuations (0–10 Å) between the apo structure and the bound structures across all chains. Paired *t*-test verified no significant difference in the fluctuations between both structures. Values obtained from the *t*-test are as follows (Table 5): chain A (*p* = 0.8287), chain B (*p* = 0.6087), chain C (*p* = 0.0520), and chain D (*p* = 0.8376. On the other hand, when the receptor is bound to benzo(ghi)perylene (Figure 10E–H), the RMSF graphs fluctuate within the range of 0–6 Å. The *p*-values for chains B (*p* = 0.0212) and C (*p* = 0.0099) indicate that the apo and bound structure is significantly different as verified by the paired *t*-test in Table 5. Figure 10F,G demonstrates the noticeable difference in the RMSF values near residues 23 and 513 for chain B, while residues 101 and 158 for chain C. Consequently, chain D (*p* = 0.2583) and chain A (*p* = 0.0546) proved otherwise. Moreover, once the TLR4 is bound to indeno(1,2,3-cd)pyrene (Figure 10I–L), minimal fluctuations in the apo and the bound structures in chains A, B, and D can be observed, in which fluctuation is greatest at 6 Å. Statistical analysis (Table 5) also verified that there are no significant differences in the fluctuations at chains A (*p* = 0.9232), B (*p* = 0.7445), and D (*p* = 0.1247). However, fluctuations in chain C are visible (*p* ≈ 0.00). Figure 10K demonstrates a noticeable difference near residue 101. These differences in RMSF values could be due to changes in internal dynamics and interaction intensities [40]. Overall, minimal fluctuations were observed once the ligand binds to the receptor.

#### 3.6.2. Molecular Dynamics Simulation of TLR4-PAH Structural Models

To better understand the binding mechanism, structural behavior, and flexibility of TLR4 protein, MDS was performed [38]. Detailed analysis of the structural fluctuations was analyzed using the 10 ns apo structure and 10 ns bound structures superimposed through MD simulations.

**Figure 11:**
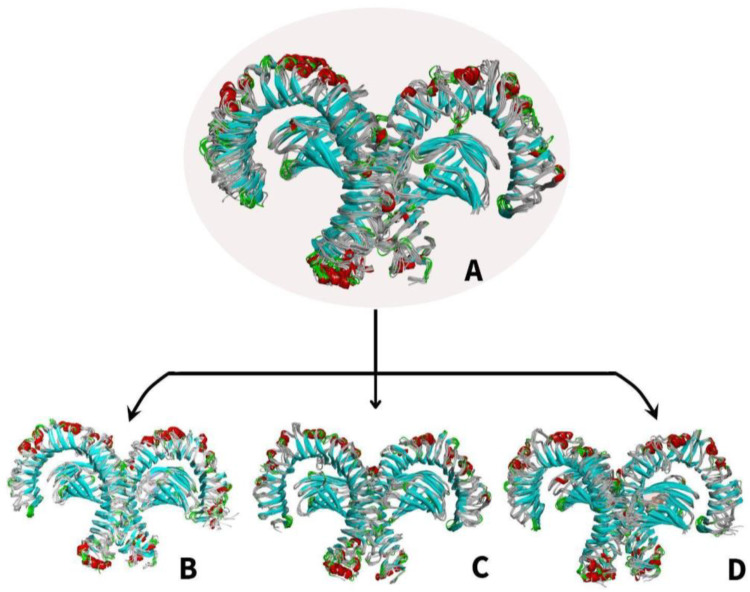
Molecular dynamics simulation comparing the apo (unbound) and bound structures of TLR4 from 0–10 ns: (**A**) superimposition of (**A**) MDS apo-TLR4 models; (**B**) TLR4-benzo[a]pyrene complexes; (**C**) TLR4-benzo(ghi)perylene complexes; and (**D**) TLR4-indeno(1,2,3-cd)pyrene complexes.

**Figure 12:**
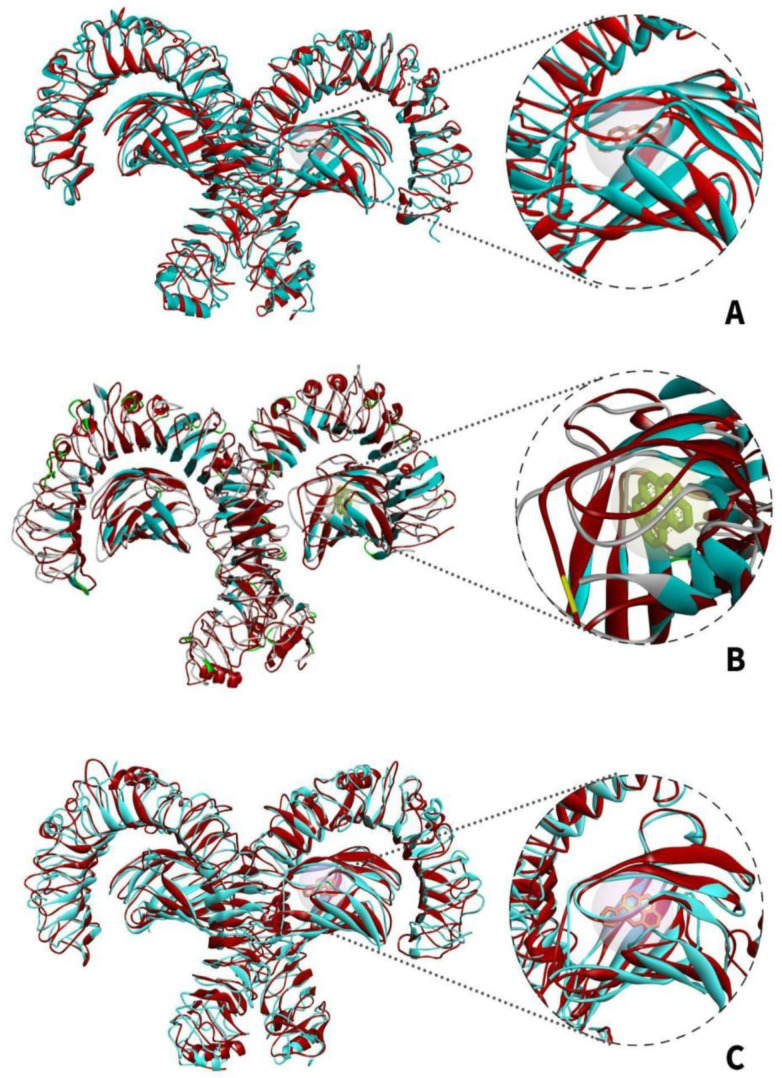
Superimposed MDS structural models of apo-TLR4 (teal) at 0 ns and TLR4-PAH complex (red) at 10 ns: (**A**) TLR4-benzo[a]pyrene; (**B**) TLR4-benzo(ghi)perylene; and (**C**) TLR4-indeno(1,2,3-cd)pyrene.

Figure 11A shows the changes in the superimposition of the apo-TLR4 structures during the 10 ns simulation while Figure 11B–D shows the changes in the superimposition of TLR4-PAH (benzo(a)pyrene, benzo(ghi)perylene, and indeno(1,2,3-cd)pyrene) complexes at 10 ns simulation, respectively. It can be observed that there are minimal changes in the structure. On the other hand, Figure 12A–C shows the changes in the general structure of apo-TLR4 at 0 ns and TLR$-PAH at 10 ns. Minimal changes can also be observed between the structures. This implies that the human TLR4 maintained its stability. Overall, the results strongly suggest that benzo(a)pyrene, benzo(ghi)perylene, and indeno(1,2,3-cd)pyrene can form a stable complex with human TLR4; therefore, these PAHs can potentially activate downstream inflammatory reaction.

### 3.7. Molecular Docking of Human TLR4 and VOC

Protein–ligand interactions happen through sub-atomic mechanics, resulting in the conformational changes in which the ligand-binding affects the protein state and its function. The relatedness of the docked structure was estimated by calculating the root mean square deviation (RMSD) [41] between the coordinates of the atoms. The least binding energy conformations were considered as the best docking pose [41]. This section is divided into four subsections that examine various interactions between the human TLR4 and VOCs.

#### 3.7.1. Interaction of Human TLR4 Residues and VOC

**Figure 13:**
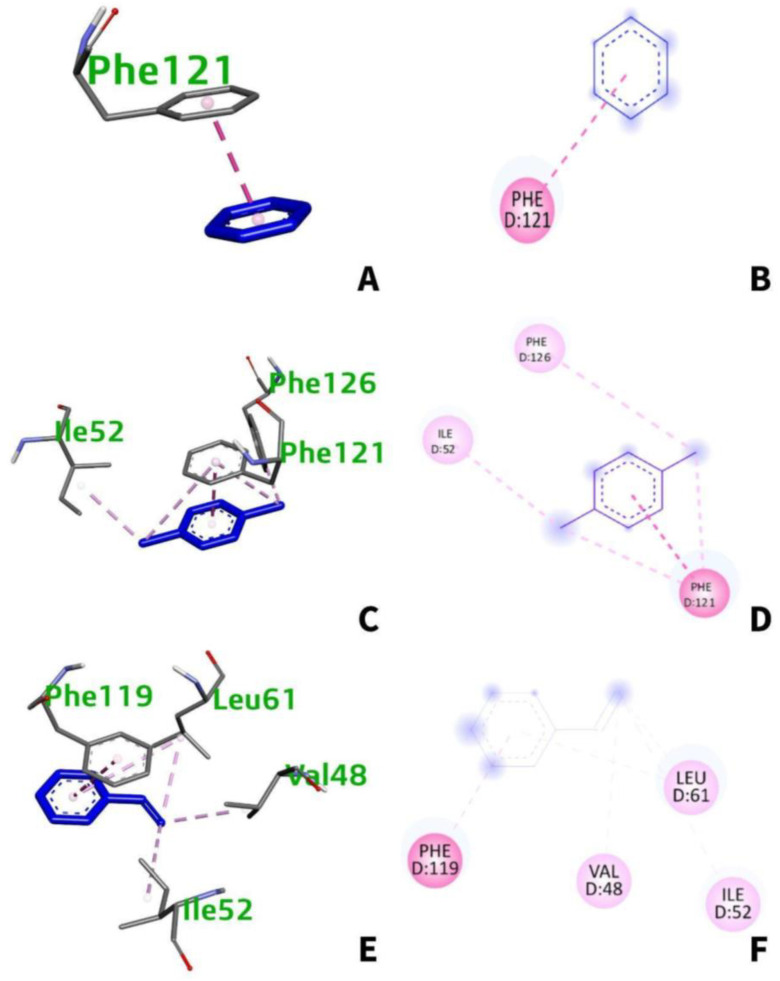
(**A**) 3D and (**B**) 2D interaction of benzene with TLR-4; (**C**) 3D and (**D**) 2D interaction of 1,4-dichlorobenzene with TLR4; (**E**) 3D and (**F**) 2D interaction of styrene with TLR4.

The structure and physical features of molecular systems are largely determined by noncovalent interactions [42]. It can be seen that four amino acids namely: Phe-119, Phe-121, Phe-126, Ile-52, Leu-61, and Val-48 of TLR4 interact with the top-binding VOCs. Hydrophobic interactions happen mostly at chain D as seen in Table 6 and the structures below (Figure 13).

It is found that only one amino acid residue interacts with benzene. Figure 13A,B shows the structure of benzene as it interacts with the amino acid residue, Phe-121. Table 6 shows that Phe-121 at chain D hydrophobically interacts with benzene at a distance of 3.722 Å through pi–pi stacking. On the other hand, three amino acids hydrophobically interact with 1,4-dichlorobenzene which can be seen in Figure 13C,D. Ile-52 and Phe-126 interact at chain D at distances of 3.742 Å and 5.131 Å, respectively, through pi–alkyl interactions. Phe-121 also interacts at chain D at a distance of 3.702 Å through pi–pi stacking (Table 6). Lastly, styrene interacts with four amino acids, namely, Phe-119, Val-48, Ile-52, and Leu-61 as seen in Figure 13E,F. All of which hydrophobically interact at chain D at certain distances through pi–alkyl interactions except for Phe-119 which interact through pi–pi stacking.

#### 3.7.2. Intermolecular Forces of Attraction

The creation of molecular complexes via intermolecular interactions is extensively investigated using the computer approach known as molecular docking. Computer-aided drug design has shown the effectiveness of molecular docking in studying molecular recognition by predicting the affinity and binding mode between the interacting molecules, such as protein–ligand interactions [43].

**Figure 14:**
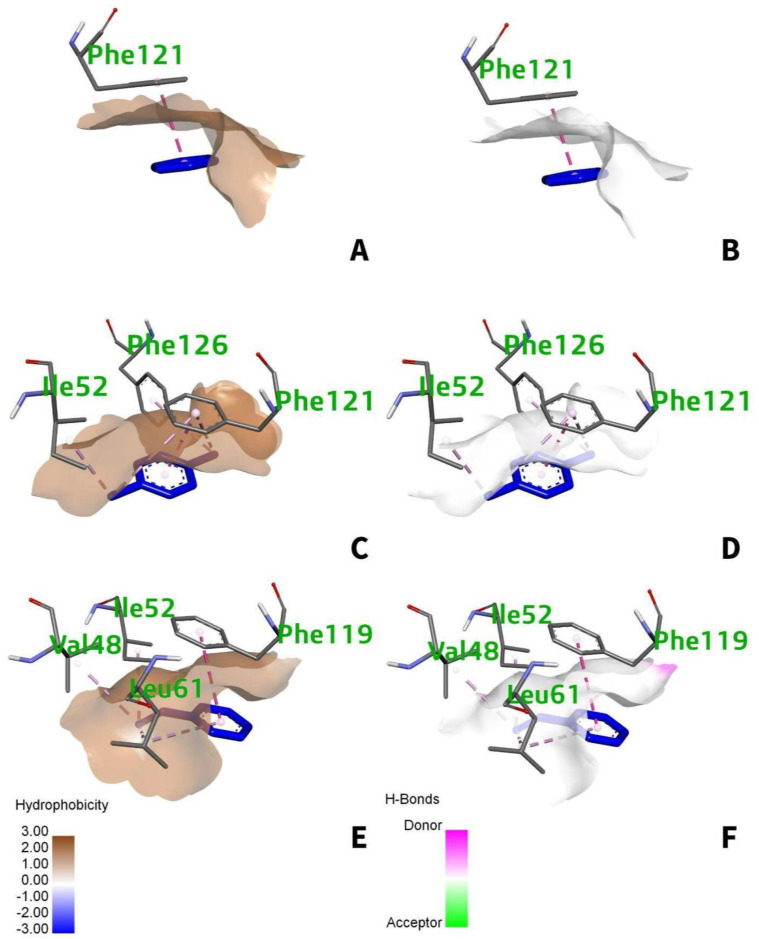
(**A**) 3D hydrophobic and (**B**) 3D H−bonding surfaces for benzene; (**C**) 3D hydrophobic and (**D**) 3D H−bonding surfaces for 1,4-dichlorobenzene; (**E**) 3D hydrophobic and (**F**) 3D H−bonding surfaces for styrene. The hydrophobicity cloud is represented by the brownish col-or, while the hydrogen donor and acceptor regions are represented by the pink and green colors, respectively.

The intermolecular forces of attraction between the human TLR4 and the individual VOCs are mostly van der Waals interactions. The benzene ring (Figure 14A) interacts hydrophobically with Phe-121. 1,4-dichlorobenzene interacts with three residues (Phe-121, Ile-52, Phe-126), while styrene interacts with four different residues (Phe-119, Leu-61, Val-48, Ile-52). All the amino acid side chains of TLR4 that interact with the ligand are hydrophobic, thus interaction is only via Van der Waals forces depicted by the hydrophobicity cloud Figure 14A,C,E). Moreover, styrene, benzene, and 1,4-dichlorobenzene indicated no hydrogen binding interactions as confirmed by the generated neutral or white molecular surfaces (Figure 14B,D,F).

#### 3.7.3. Ramachandran Plot

When determining whether or not a model is legitimate, it is necessary to take a number of factors into consideration, including the interactions between surrounding residues, the atoms that are involved, and the positions of the residues. The stereochemical qualities of the model, including bond angles, bond lengths, correct chirality, and ring structure, along with other geometric factors, need to be validated. These attributes are included in the model. The Ramachandran plot is a plot in which the torsional angles of the amino acids phi and psi in a protein sequence are shown in a two-dimensional format (2D). In order to determine the conformation of the peptide, the values are plotted against one another, and the angular spectrum ranges between 180 and +180 degrees on both the x- and y-axes. The Ramachandran outlier is a depiction of those amino acids that reside in areas of the plot that are not favorable [44].

**Figure 15:**
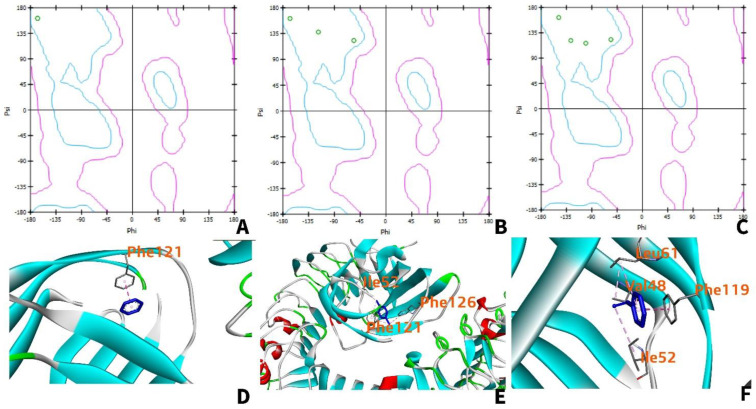
Ramachandran plots of TLR4 amino acids interacting with benzene (**A**), 1,4-dichlorobenzene (**B**), and styrene (**C**). The green circles indicate the amino acid residues interacting with the ligand. The teal regions are indicative of favorable regions while the areas enclosed by the teal and pink regions are allowed but rare due to the torsion angles. Beyond the pink region, residues are not allowed due to the possibility of collision; 3D interaction of TLR4 with benzene (**D**), 1,4−dichlorobenzene (**E**), and styrene (**F**).

In the Ramachandran plot of TLR-4 amino acids interacting with benzene, 1,4−dichlorobenzene, and styrene (Figure 15A,C,E), all the amino acid residues are within the favorable region. The benzene ring (Figure 15D) prefers to interact hydrophobically with the Phe-121 amino acid side chain. Additionally, the 1,4-dichlorobenzene interacts with three hydrophobic side chains, while styrene (Figure 15F) interacts with four different amino acid residues. All the amino acid residues are in beta-pleated sheet conformation as represented by the teal regions in the TLR4.

#### 3.7.4. TLR4 Amino Acids Interacting with LPS and VOCs

It is possible to depict the three-dimensional structure of a protein as a graph, with nodes standing in for residues, and edges denoting the strength of non-covalent interactions. Depending on where the amino acids are located in the basic structure, these protein contact networks may be divided into long-range interaction networks and short-range interaction networks [45].

**Figure 16:**
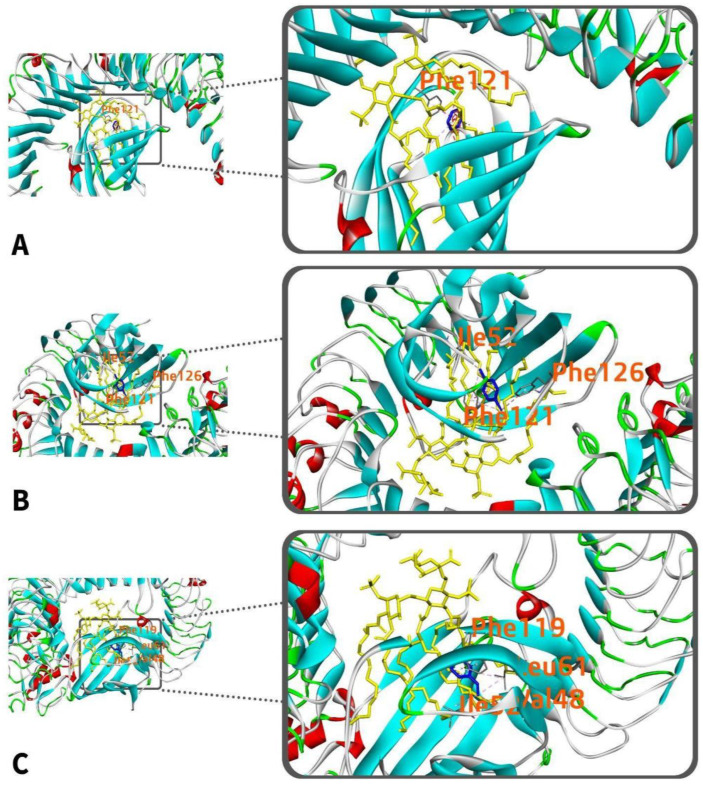
Superimposed interactions of benzene (**A**), 1,4-dichlorobenzene (**B**), and styrene (**C**) with human TLR4-LPS complex. The PAH is represented by the blue molecule, while the LPS is represented by the yellow molecule.

As seen in Figure 16, TLR4 interacts with benzene through a single amino acid residue, Phe-121. In comparison, LPS and TLR4 have a hydrophobic interaction at 40 amino acid residues. Only Phe-121 is shared by benzene and LPS. Phe-121 interacts hydrophobically with chain D at a distance of 3.974 Å, although it does so through pi–alkyl interactions rather than pi–pi stacking, as seen in Table 7. On the other hand, TLR4 interacts hydrophobically with 1,4-dichlorobenzene at three amino acid residues: Phe-121, Ile-52, and Phe-126. 1,4−dichlorobenzene and LPS have only one common interacting amino acid residue, Phe-121. Additionally, Phe-121 interacts hydrophobically through pi–alkyl interaction at a distance of 3.974 Å. TLR4, on the other hand, interacts with styrene at four distinct amino acids: Phe-119, Leu-61, Val-48, and Ile-52. Styrene and LPS, nevertheless, share just one common amino acid residue, Phe-119. This happens through pi–alkyl contact hydrophobically at a distance of 5.369 Å (Table 7).

### 3.8. Molecular Dynamics Simulation of TLR4-VOC Complexes

To further evaluate the reliability of the docked ligand–protein complexes in 3D coordinates of the best docking configuration, TLR4 with its bound VOCs was subjected to MDS [46]. CABS-Flex 2.0 was utilized to determine the amino acid residues with the highest degree of flexibility [38]. Moreover, this section is divided into two subsections where further examination of the RMSF graphs and molecular dynamics are discussed.

#### 3.8.1. RMSF of Apo-TLR4 and TLR4-VOC Complexes

Higher RMSF values are indicative of the high residue flexibility [38] while lower values correspond to more stable ligand binding [33]. The plotted values for the RMSF for Apo-TLR4 and TLR4-VOC complexes were generated via MATLAB R2021a.

**Figure 17:**
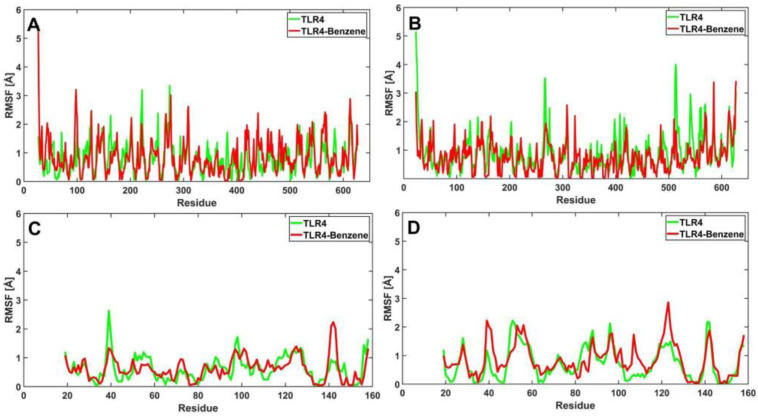

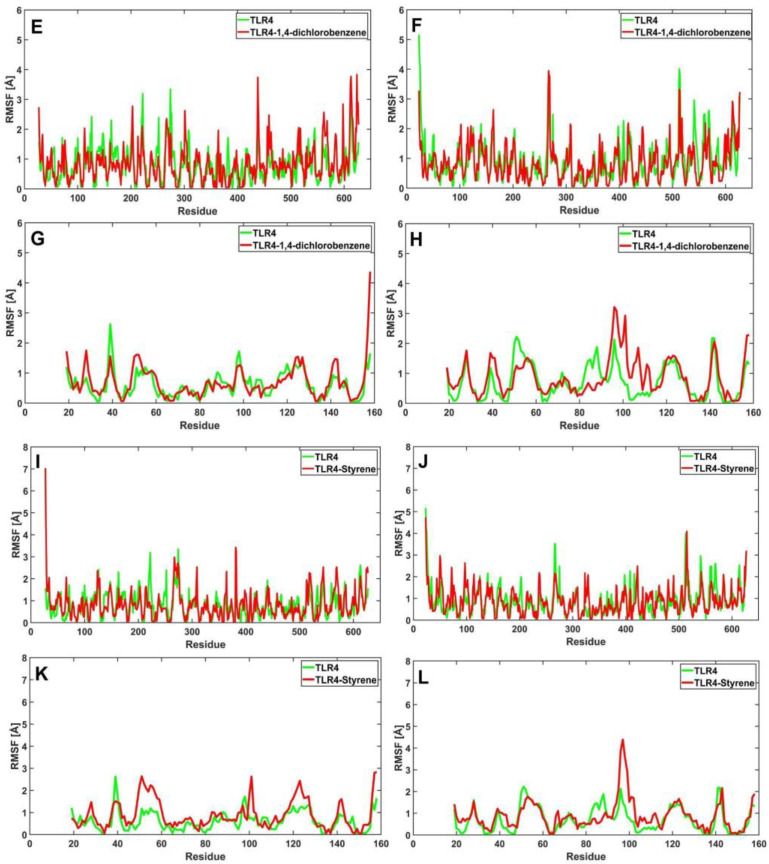
RMSF profiles for the amino acid residues of the apo (green) and bound (red) structures of TLR4: TLR4 vs. TLR4-benzene chains A–D (**A**–**D**); TLR4 vs. TLR4-1,4-dichlorobenzene chains A–D (**E**–**H**); and TLR4 vs. TLR4-styrene chains A–D (**I**–**L**).

For TLR4 bound to benzene (Figure 17A–D), minimal fluctuations (0–6 Å) were observed for chains A and C. Paired *t*-test verified there are no significant differences as the *p*-values are 0.1761 and 0.8625 for chains A and C, respectively (Table 8). For chains B and D, fluctuations were observed with *p*-values of ≈0.00 and 0.0362, respectively. Additionally, Figure 17B,D demonstrates the noticeable differences in RMSF values near residues 23, 267, and 513 for chain B, while for chain D near residues 39 and 142. On the other hand, when the receptor is bound to 1,4-dichlorobenzene (Figure 17E–H), minimal fluctuations (0–6 Å) were observed for chains A to C with *p* values of 0.2437, 0.4919, and 0.2096 for chains A, B, and C, respectively. For chain D, fluctuations are more visible (*p* = 0.0293). This is visible in Figure 17H, in which fluctuations occur near residues 96 and 107. Lastly, for TLR4 bound to styrene, minimal fluctuations were visible in chains A (*p* = 0.6545) and B (*p* = 0.9934). In chains C and D, fluctuations were noticeable with maximum fluctuation reaching 7 Å with *p* values of ≈0.00 and 0.0210, respectively. Figure 17K–L, shows the noticeable differences near residues 51 and 101 for chain C, and residue 97 for chain D. Overall, minimal fluctuations are observed once the ligands bind to the receptor. Table 8 summarizes all the generated *p* values.

#### 3.8.2. Molecular Dynamics Simulation of TLR4-VOC Structural Models

The concept of binding mechanism, structural behavior, and flexibility of the protein complex can be better understood through molecular dynamics simulations [38]. Detailed analysis of the fluctuations was analyzed wherein the time was set at 0–10 ns for each system.

**Figure 18:**
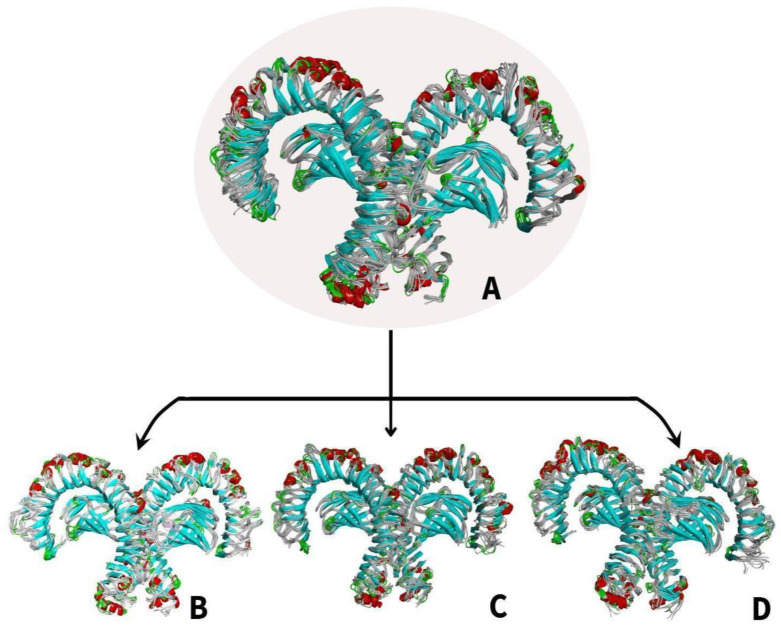
Molecular dynamics simulation comparing the apo (unbound) and bound structures of TLR4 from 0–10 ns: (**A**) superimposition of (**A**) MDS apo-TLR4 models; (**B**) TLR4-benzene complexes; (**C**) TLR4-1,4-dichlorobenzene complexes; and (**D**) TLR4-styrene com-plexes.

**Figure 19:**
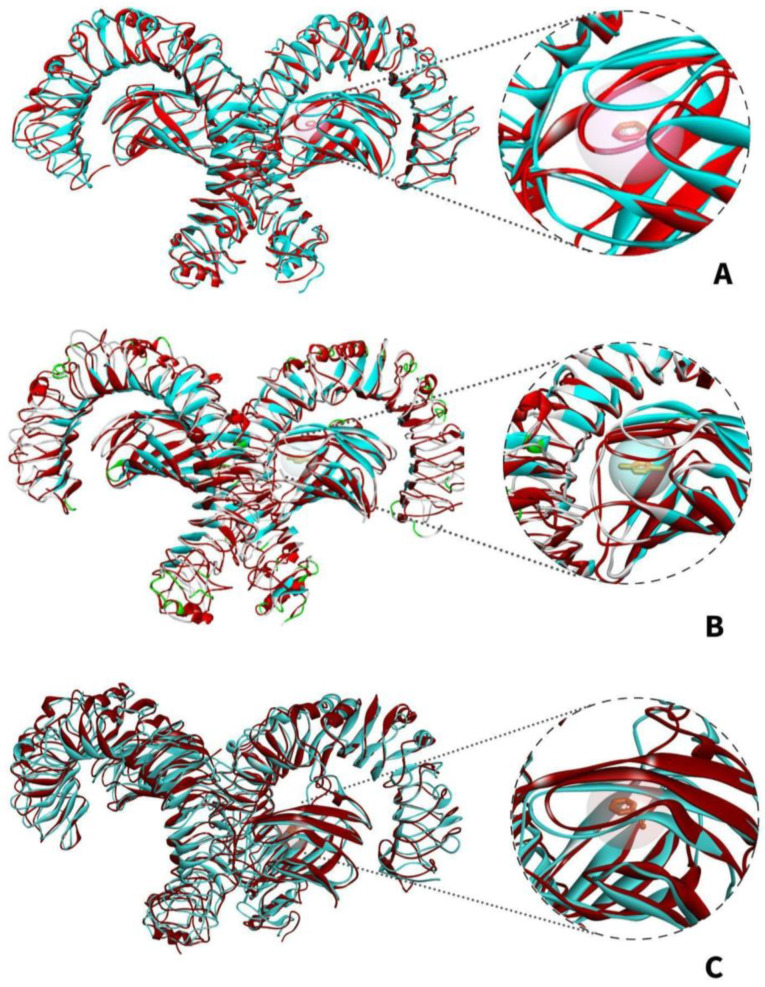
Superimposed MDS structural models of apo-TLR4 (teal) at 0 ns and TLR4-PAH complex (red) at 10 ns: (**A**) TLR4-benzene; (**B**) TLR4-1,4-dichlorobenzene; and (**C**) TLR4-styrene.

The models Figure 18 depict the superimposed structures apo-TLR4 and TLR4-VOC complexes from 0 to 10 ns. There were minimal changes in the general structures of TLR4 bound to benzene, 1,4-dichlorobenzene, and styrene compared with apo-TLR4 (Figure 18B–D). Furthermore, discerning the structure of apo-TLR4 at 0 ns superimposed with the bound structure at 10 ns in Figure 19A–C, only minimal changes in the protein structure were visually noticeable. The results of the molecular dynamics suggest that benzene, 1,4-dichlorobenzene, and styrene can form a stable complex with the TLR4, hence can be agents that can potentially induce pro-inflammatory reactions.

## 4. Discussion

### 4.1. Role of Polycyclic Aromatic Hydrocarbons in Inducing Cancer

From Table 1, benzo(a)pyrene, benzo(ghi)perylene, and indeno(1,2,3,-cd)pyrene were recorded with the top three highest binding affinities among the 14 PAHs. The highest recorded binding affinity of –10.0 kcal/mol belonged to indeno(1,2,3,-cd)pyrene. Benzo(ghi)perylene exhibited an affinity of –9.0 kcal/mol and benzo(a)pyrene afforded –8.9 kcal/mol. In comparison to the binding affinity of LPS to TLR4 which is –4.1 kcal/mol, the binding affinities of the PAHs are considerably higher which indicates that they are more likely to induce inflammation. Collectively, the three PAHs are truly carcinogenic to humans as benzo(a)pyrene is one of the most carcinogenic members of the PAH family [47]. Indeno(1,2,3-cd)pyrene is known to be the most prominent PAH associated with PM_2.5_ [48] and is one of the carcinogenic compounds found in high concentrations in the atmospheres of metro cities [49]. Benzo(a)pyrene is commonly linked to causing chest pain, cough, rashes, respiratory problems, and low immunity after prolonged exposure. While exposure to indeno(1,2,3,-cd)pyrene can induce chronic health effects which can last for months to years, it was proven to cause skin and lung cancer, as well as induce potential reproductive damage in animal studies. On the other hand, benzo(ghi)perylene is classified as moderately carcinogenic to humans. Studies have found benzo(ghi)perylene to induce liver cancer, precancerous lesions, and precancerous stomach lesions with rates of 21.1, 68.4, and 78.9 percent, respectively [50].

### 4.2. Role of Volatile Organic Compounds in Inducing Cancer

Among the 10 VOC carcinogens analyzed, benzene, 1,4-dichlorobenzene, and styrene have the most negative binding affinity. Using the binding affinity (−4.1 kcal/mol) of the LPS obtained for the Autodock Vina’s scoring function as a standard to determine the VOCs’ proinflammatory capacity, benzene having the binding affinity of −3.6 kcal/mol and 1,4-dichlorobenzene with a binding affinity of −3.9 kcal/mol, can only be possibly less proinflammatory to humans. However, the case is not the same for styrene which has a binding affinity of −4.6 kcal/mol. As compared to the binding affinity of the LPS, styrene is a possible potent compound in inducing inflammation.

According to the United States Environmental Protection Agency [51], volatile organic compounds are those with a high vapor pressure but a poor solubility in water. Numerous VOCs are synthetic substances generated by humans and employed in the creation of paints, medicines, and refrigerants. In this study, the following VOCs were examined: benzene, 1,4-dichlorobenzene, and styrene. Benzene, an aromatic hydrocarbon, is a prevalent air pollutant that originates from human activities such as burning. It is present in a wide variety of products, including gasoline, automobile exhaust, and tobacco smoke. Since 1979, benzene has been classified as carcinogenic to humans from the fundamental evidence that it causes leukemia [52]. In animal bioassays, 1,4-dichlorobenzene was also shown to trigger the formation of liver cancer [53]. Styrene, on the other hand, is a vital and common component of waxes, paints, plastics, and resins [54]. According to the National Institute of Environmental Health Sciences, there are few human studies that demonstrate that a high styrene concentration may raise the risk of lymphohematopoietic malignancies, such as leukemia and lymphoma, as well as genetic damage in white blood cells [55].

## 5. Conclusions

Virtual screening utilizing molecular docking algorithms has grown in popularity as a method for developing novel medications and deciphering molecular mechanisms due to time and financial savings associated with pre-analyses using in silico drug screening compared to direct conventional laboratory research. In this study, we used a computational protein–ligand docking approach and modeled interactions between selected PAHs and VOCs and human TLR4.

Out of the 14 PAHs analyzed, benzo(a)pyrene, benzo(ghi)perylene, and indeno(1,2,3,-cd)pyrene had the highest binding affinity and therefore can be considered highly carcinogenic due to their potential proinflammatory capacities. Consequently, out of the 10 VOCs studied, benzene, 1,4-dichlorobenzene, and styrene obtained the highest binding affinities for potential induction of the TLR4 inflammatory pathway. More in vitro and in vivo studies (e.g., lipophilicity, hydrophilicity, logP, IC_50_, pro-inflammatory induction in macrophages) are required to corroborate these in silico findings.

For the molecular docking simulation, the RMSF graphs and superimposed models of apo-TLR4 and TLR4 complexed with PAHs and VOCs confirmed that the structure of TLR4 was stable. 

It is possible that in silico studies can be utilized in conjunction with clinical experiments to help guide the creation of more precise hypotheses and experimental research regarding the possible effects of PAHs and VOCs in inducing cancer. Preliminary information can be gathered using molecular docking, molecular dynamics, and in silico experiments, which then can be used to guide research in future in vivo and in vitro studies. In multidisciplinary research, this type of computational tool can help researchers and doctors modify their experimental plans in an effort to reduce expenses and improve research efficiency.

In future studies, in vitro studies are necessary to ascertain the potential pro-inflammatory effects of other PAHs and VOCs in relation to the human TLR4 molecule, which could also lead to the determination of its toxicity and carcinogenicity. For the development of the TLR4 signaling modulators, it is recommended to analyze the effects of the PAHs and VOCs based on the production of inflammatory cytokines. Lastly, different carcinogenic pollutants may also be tested to compare their effects against the PAHs and VOCs mentioned in this study.

## Figures and Tables

**Table 1:** Binding affinities of PAHs to human TLR4 generated by Autodock Vina.

PAH	Affinity (kcal/mol)	Carcinogenicity
indeno(1,2,3-cd)pyrene	−10.0	Highly carcinogenic
Benzo(ghi)perylene	−9.0	Moderately carcinogenic
Benzo[a]pyrene	−8.9	Highly carcinogenic
Benzo [k] fluoranthene	−8.9	Moderately carcinogenic
Chrysene	−8.2	Moderately carcinogenic
Benzo [a] anthracene	−8.1	Moderately carcinogenic
Pyrene	−7.4	Highly carcinogenic
Fluoranthene Anthracene	−7.3 −6.8	Weak carcinogenic Weak carcinogenic
Phenanthrene	−6.8	Highly carcinogenic
Fluorene	−6.4	Weak carcinogenic
Acenaphthylene	−6.2	Weak carcinogenic
Acenaphthene	−6.0	Weak carcinogenic
Naphthalene	−5.5	Weak carcinogenic

**Table 2:** Binding affinities of VOCs to human TLR4 generated by Autodock Vina.

VOC	Affinity (kcal/mol)	Carcinogenicity
Styrene	−4.6	Possibly carcinogenic to humans
1,4-dichlorobenzene	−3.9	Possibly carcinogenic to humans
Benzene	−3.6	Carcinogenic to humans
1,3-butadiene	−3.1	Probably carcinogenic to humans
Tetrachloroethylene	−3.0	Probably carcinogenic to humans
Trichloroethylene	−2.7	Probably carcinogenic to humans
Chloroform	−2.6	Possibly carcinogenic to humans
1,2-dichloroethane	−2.3	Possibly carcinogenic to humans
Vinyl chloride	−2.2	Carcinogenic to humans
Methylene chloride	−2.1	Possibly carcinogenic to humans

**Table 3:** Interaction of amino acid residues of TLR4 to benzo[a]pyrene, benzo(ghi)perylene, and indeno (1,2,3-cd)pyrene.

PAH	Name	Distance Å	Category	Types
Benzo(a)pyrene	Phe-119 (D)	5.336 4.262		Pi–pi Stacking
	Phe-121 (D)	4.082 4.167 5.173 3.660	Hydrophobic	Pi–pi Stacking
	Ile-52 (D)	4.846	Hydrophobic	Pi–alkyl
	Leu-61 (D)	5.299	Hydrophobic	Pi–alkyl
Benzo(ghi)perylene	Leu-61 (D)	3.702 3.963	Hydrophobic	Pi–sigma
	Phe-119 (D)	5.163 5.866	Hydrophobic	Pi–pi T shaped
	Val-48 (D)	4.984 5.375	Hydrophobic	Pi–alkyl
	Ile-52 (D)	5.313 5.408	Hydrophobic	Pi–alkyl
	Val-135 (D)	4.935	Hydrophobic	Pi–alkyl
Indeno(1,2,3-cd)pyrene	Ile-52 (D)	3.892 3.390 3.441 3.748	Hydrophobic	Pi–sigma
	Phe-121(D)	4.142 4.649	Hydrophobic	Pi–pi Stacking
	Phe-151(D)	5.840	Hydrophobic	Pi–pi T shaped
	Leu-61 (D)	5.180	Hydrophobic	Pi–alkyl
	Ile-32 (D)	5.464	Hydrophobic	Pi–alkyl
	Val-48 (D)	5.173	Hydrophobic	Pi–alkyl

Phe—phenylalanine; Ile—isoleucine; Leu—leucine; Val—valine.

**Table 4:** Interactions of common amino acid residues between TLR4 and LPS with interactions of benzo[a]pyrene, benzo(ghi)perylene, and indeno(1,2,3-cd)pyrene.

PAH	Name	Distance Å	Category	Types
Benzo[a]pyrene-LPS & TLR-4	Phe-119 (D)	5.369	Hydrophobic	Pi–alkyl
	Phe-121 (D)	3.974	Hydrophobic	Pi–alkyl
Benzo(ghi)perylene-LPS & TLR-4	Phe-119 (D)	5.369	Hydrophobic	Pi–alkyl
	Val-135 (D)	4.850	Hydrophobic	Pi–alkyl
Indeno(1,2,3-cd)pyrene-LPS & TLR-4	Phe-121 (D)	3.092	Hydrophobic	Pi–sigma
	Phe-151 (D)	3.840	Hydrophobic	Pi–sigma

Phe—phenylalanine; Val—valine.

**Table 5:** Paired *t*-test (*p* values) of RMSF between apo-TLR4 and TLR4-PAH complexes.

PAH	Chain A	Chain B	Chain C	Chain D
Benzo[a]pyrene	0.8287	0.6087	0.0520	0.8376
Benzo(ghi)perylene	0.0546	0.0212	0.009	0.2583
Indeno(1,2,3-cd)pyrene	0.9232	0.7445	0.00	0.1247

**Table 6:** Interaction of amino acid residues of TLR4 to benzene, 1,4-dichlorobenzene, and styrene.

PAH	Name	Distance Å	Category	Types
Benzene	Phe-121(D)	3.722	Hydrophobic	Pi–pi Stacking
1,4-dichlorobenzene	Phe-121 (D)	3.702	Hydrophobic	Pi–pi Stacking
	Ile-52 (D)	3.742	Hydrophobic	Pi–alkyl
	Phe-126 (D)	5.131	Hydrophobic	Pi–alkyl
Styrene	Phe-119 (D)	4.306	Hydrophobic	Pi–pi Stacking
	Val-48 (D)	3.993	Hydrophobic	Pi–alkyl
	Ile-52 (D)	5.069	Hydrophobic	Pi–alkyl
	Leu-61 (D)	3.975 5.492	Hydrophobic	Pi–alkyl

Phe—phenylalanine; Ile—isoleucine; Leu—leucine; Val—valine.

**Table 7:** Interactions of common amino acid residues between TLR4 and LPS with interactions of benzene, 1,4−dichlorobenzene, and styrene.

PAH	Name	Distance Å	Category	Types
Benzene—LPS & TLR4	Phe-121 (D)	3.974	Hydrophobic	Pi–alkyl
1,4-dichlorobenzene—LPS & TLR4	Phe-121 (D)	3.974	Hydrophobic	Pi–alkyl
Styrene—LPS & TLR4	Phe-119 (D)	5.369	Hydrophobic	Pi–sigma

Phe—phenylalanine.

**Table 8:** Paired *t*-test (*p* values) of RMSF between apo-TLR4 and TLR4-VOC complexes.

VOC	Chain A	Chain B	Chain C	Chain D
Benzene	0.1761	0.00	0.8625	0.0362
1,4-dichlorobenzene	0.2437	0.4919	0.2096	0.0293
Styrene	0.6545	0.9934	0.00	0.0210
